# The impact of ferroptosis and ferroptosis-related non-coding RNAs on breast cancer progression

**DOI:** 10.3389/fcell.2024.1506492

**Published:** 2024-12-23

**Authors:** Wenhui Liu, Chenjun Jiang, Yun Ma, Wentao Wang, Jing Peng, Weiqing Ma, Shuxin Xu, Duoming Wu

**Affiliations:** ^1^ The First Clinical Medical College, Lanzhou University, Lanzhou, China; ^2^ Department of Breast Surgery, The First Hospital of Lanzhou University, Lanzhou, Gansu, China

**Keywords:** breast cancer, ferroptosis, noncoding RNAs, miRNAs, CircRNAs, lncRNAs

## Abstract

Ferroptosis, distinct from apoptosis, is primarily characterized by the accumulation of iron-dependent lipid peroxides (LPO) and reactive oxygen species (ROS). This process plays a pivotal role in the pathophysiology of various diseases and has recently emerged as a promising therapeutic strategy in oncology, garnering significant attention. Non-coding RNAs (ncRNAs), including microRNAs (miRNAs), long non-coding RNAs (lncRNAs), and circular RNAs (circRNAs), serve as crucial regulators in numerous biological processes, particularly in cancer initiation and progression. Increasing research efforts are focused on targeting ferroptosis through modulation of these ncRNAs. This review provides an overview of the mechanisms underlying ferroptosis and explores the roles of ncRNAs in breast cancer (BC) and its regulation. Furthermore, we examine the interactions between ferroptosis and ncRNAs in BC, aiming to identify potential therapeutic targets for BC treatment.

## 1 Introduction

Breast cancer (BC) represents a significant global health threat to women (Makhoul et al., 2018). Recent cancer statistics indicate that BC has surpassed lung cancer as the most prevalent malignancy among women (Nik-Zainal et al., 2016). Although BC can occur post-adolescence, its incidence increases with age (Duffy et al., 2012). Despite significant advancements in cancer diagnosis, treatment, and prevention, the global mortality rate from BC remains the fifth highest among all cancers, primarily due to the growing resistance to chemotherapy and radiotherapy (Sung et al., 2021). Approximately 90% of BC-related deaths are attributed to distant metastases from recurrent or primary tumors. Consequently, early diagnosis, prompt treatment, and the development of more effective therapeutic strategies are crucial for improving patient outcomes (Chen et al., 2018).

Ferroptosis, a novel form of regulated cell death, was first identified in 2012 (Dixon, 2017).

It is characterized by the accumulation of iron-dependent lipid peroxides (LPO) and reactive oxygen species (ROS) (Huang R. et al., 2023). However, this unique, iron-dependent mode of cell death—distinct from apoptosis, autophagy, and necrosis—had been observed prior to its formal designation. Morphologically, ferroptotic cells exhibit shrunken mitochondria, increased membrane density, and reduced cristae, while nuclear morphology remains relatively unaffected (Mou et al., 2019; Dixon et al., 2012; Li J. et al., 2020). Ferroptosis plays a pivotal role in various physiological and pathological processes, including neurodegenerative diseases, cancer, and cardiovascular disorders (Li J. et al., 2020). Interestingly, tumor cells resistant to conventional therapies still exhibit high sensitivity to ferroptosis (Hassannia et al., 2019). Therefore, understanding the mechanisms and regulation of ferroptosis is crucial for elucidating the pathogenesis of these diseases and developing novel therapeutic strategies. However, the interactions between ferroptosis and BC remain underexplored, and the impact of ferroptosis on BC prognosis is not yet fully understood.

Non-coding RNAs (ncRNAs) are RNA molecules that do not encode proteins but possess regulatory functions. These include microRNAs (miRNAs), long non-coding RNAs (lncRNAs), and circular RNAs (circRNAs). ncRNAs play crucial roles in various biological processes, particularly in regulating cancer initiation and progression. Recent evidence suggests that ncRNAs can modulate ferroptosis by regulating ferroptosis-related genes and metabolic pathways. Through these mechanisms, ncRNAs may either promote or inhibit cancer development (Luo Y. et al., 2021).

This review discusses the mechanisms underlying ferroptosis and examines its relationship with BC. Additionally, we review the structure and function of ncRNAs and their roles in both BC and ferroptosis. Furthermore, we summarize ferroptosis-related ncRNAs in BC that are crucial for anticancer therapy, offering new insights into the development of novel therapeutic strategies for BC. Finally, we explore recent advancements and future prospects of targeting ferroptosis-related ncRNAs in BC treatment.

## 2 Ferroptosis

### 2.1 Mechanisms of ferroptosis

**FIGURE 1:**
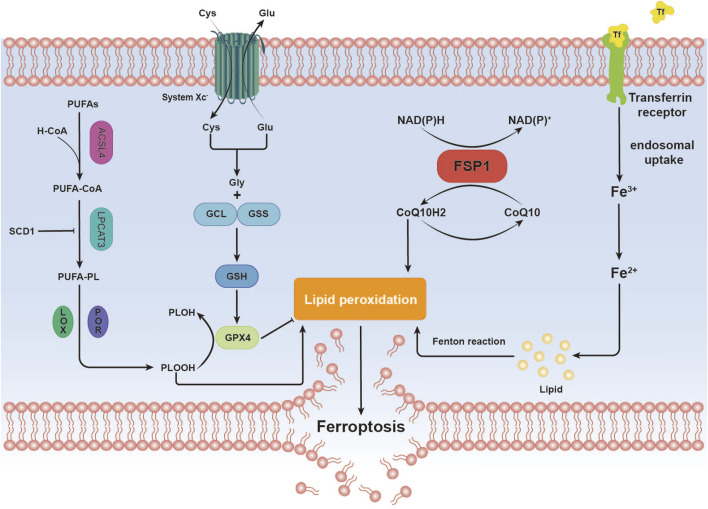
Overview of Ferroptosis Mechanisms. This image illustrates the mechanistic pathway of ferroptosis, a distinctive form of cell death characterized by iron-dependent lipid peroxidation. Iron (Fe) Metabolism. Polyunsaturated Fatty Acids (PUFAs) Metabolism. Antioxidant Defense. Lipid Peroxidation. These steps elucidate the mechanistic basis by which ferroptosis, through iron-dependent lipid peroxidation, culminates in the destruction of cellular membranes, leading to cell death.

Ferroptosis, an iron-dependent form of regulated cell death distinct from apoptosis, is closely associated with disruptions in redox homeostasis. This process is triggered by an imbalance in redox regulation (Dixon and Olzmann, 2024). Key features of ferroptosis include the excessive accumulation of ROS and LPO, often driven by iron overload or lipoxygenase activity. The loss or reduced expression of critical antioxidant enzymes, such as glutathione peroxidase 4 (GPX4), further exacerbates lipid peroxidation, ultimately leading to cell death (Jiang et al., 2021) (Figure 1).

### 2.2 Oxidative system overactivation

Iron is an essential element in biological systems, playing key roles in oxygen transport, electron transfer, and enzymatic reactions (Morales and Xue, 2021). Within cells, iron primarily exists as ferrous (Fe^2^⁺) and ferric (Fe³⁺) ions (Fairweather-Tait, 2001). Under normal conditions, cells maintain iron homeostasis through tightly regulated mechanisms that govern iron uptake, storage, utilization, and export (Liu et al., 2022). However, in pathological conditions, such as genetic mutations, environmental toxin exposure, or disease states, this balance can be disrupted, leading to iron accumulation, oxidative stress, and the initiation of ferroptosis (Nakamura et al., 2019). Excessive accumulation of ferrous ions triggers the Fenton reaction, generating highly reactive hydroxyl radicals (·OH) and other ROS (Toyokuni, 2002). These ROS then target and damage various intracellular biomolecules, including lipids, proteins, and nucleic acids (Zhang K. et al., 2020).

Acyl-CoA synthetase long-chain family member 4 (ACSL4) is a key enzyme in lipid metabolism (Doll et al., 2017; Zou et al., 2019), critical for initiating lipid peroxidation and determining cellular susceptibility to ferroptosis (Cui et al., 2021). ACSL4 selectively activates specific polyunsaturated fatty acids (PUFAs), such as arachidonic acid (AA) and adrenic acid, by converting them into acyl-CoA derivatives (Xiang et al., 2024). This modification alters membrane lipid composition, increasing their susceptibility to peroxidation and promoting ferroptosis (Doll et al., 2017; Yuan et al., 2016). ACSL4-mediated incorporation of PUFAs into membrane phospholipids further enhances their vulnerability to oxidative damage (Chen X. et al., 2021).

Lipoxygenases (LOXs), a family of non-heme iron-containing dioxygenases, directly oxidize PUFAs within cellular membranes (Kuhn et al., 2018), particularly AA and adrenic acid (Gaschler and Stockwell, 2017). Due to their multiple double bonds, PUFAs are optimal substrates for LOX-mediated oxidation, leading to the generation of various LPO (Porter et al., 1979; Xie LH. et al., 2022).

### 2.3 Imbalance of antioxidant systems

Glutathione (GSH), a tripeptide composed of glutamate, cysteine, and glycine, is a critical intracellular antioxidant (Niu et al., 2021). Under normal conditions, GSH collaborates with GPX4 to inhibit ferroptosis. GPX4, a selenium-dependent enzyme, is the primary catalyst responsible for reducing phospholipid hydroperoxides (PLOOHs) to their corresponding alcohols in mammalian cells, thereby mitigating lipid peroxidation and protecting cell membranes from oxidative damage (Ursini et al., 1982; Ursini et al., 1985). Disruption of the GSH system can occur through several mechanisms:

First of all, GSH synthesis is a key determinant of GSH system function. This process is contingent on the cellular availability of cysteine, which is primarily imported into cells via the cystine/glutamate antiporter system (system Xc-) (Dixon et al., 2012; Liu et al., 2022). Dysfunction of the Xc-system reduces cysteine uptake, impairing GSH synthesis. In the absence of GSH, GPX4 loses its ability to detoxify LPO, leading to increased lipid peroxidation (Liu et al., 2022).

Secondly, increased GSH consumption disrupts the balance of GSH availability. Under pathological conditions, reduced GSH is required to neutralize enhanced cellular activities, such as iron accumulation, elevated lipoxygenase expression, increased enzyme activity, and excessive LPO (Ursini and Maiorino, 2020). During the reduction of LPO to their corresponding alcohols, GPX4 utilizes its selenocysteine residues to transfer two electrons—typically provided by GSH—to the LPO. In some cases, these electrons may also be sourced from other low-molecular-weight thiols or protein thiols (Maiorino et al., 2018). This reaction occurs as GSH binds to the active site of GPX4 via its thiol group, facilitating the reduction of the peroxyl bond in the lipid peroxide (Chen X. et al., 2021). Additionally, exogenous compounds or metabolic byproducts may bind to GSH, accelerating its depletion. For example, RSL3 has been shown to directly inhibit GPX4, destabilizing the GSH system and impairing antioxidant defense (Jiang et al., 2021).

Thirdly, reduced GSH regeneration: After its antioxidant activity, GSH is oxidized to glutathione disulfide (GSSG). Normally, intracellular glutathione reductase uses nicotinamide adenine dinucleotide phosphate (NADPH) as a reducing agent to convert GSSG back into GSH, thereby maintaining cellular GSH levels (Flohé, 2013). However, when glutathione reductase activity is suppressed or NADPH availability is limited, GSSG cannot be efficiently recycled to GSH, resulting in a decline in the pool of active GSH (Averill-Bates, 2023).

While the GPX4 pathway serves as a major antioxidant defense in ferroptosis, studies have identified ferroptosis suppressor protein 1 (FSP1) as another critical regulator of ferroptosis inhibition (Bersuker et al., 2019; Doll et al., 2019). Initially considered a pro-apoptotic gene (Wu et al., 2002), FSP1 was later found to confer resistance to ferroptosis in cells lacking GPX4. CoQ10, a lipid-soluble quinone compound, is abundant in mitochondria and cellular membranes, where it plays a key role in both antioxidant defense and energy metabolism (Raizner, 2019). CoQ10 exists in two redox states: reduced and oxidized forms. Ubiquinol scavenges lipid peroxyl radicals, effectively neutralizing lipid peroxidation and thus preventing ferroptosis. FSP1 facilitates the regeneration of reduced CoQ10, ensuring a continuous supply of this antioxidant. When the CoQ10 system is dysregulated, ubiquinol levels decrease, ubiquinone levels rise, and the risk of ferroptosis escalates (Doll et al., 2019). Dysregulation of CoQ10 can occur through several mechanisms:

#### 2.3.1 Decreased synthesis of CoQ10

CoQ10 is synthesized in various tissues and primarily stored in mitochondria. Its biosynthesis involves multiple enzymatic steps, and disruptions at any stage, due to genetic defects or pathological conditions, can lead to insufficient CoQ10 production (Raizner, 2019).

#### 2.3.2 Increased consumption of ubiquinol

During oxidative stress, high levels of ROS and lipid hydroperoxides (LOOH) are produced (Gordan et al., 2018; Berdoukas et al., 2015; Hershko, 2010; Kruszewski, 2003), which rapidly oxidize ubiquinol to ubiquinone, depleting the active antioxidant form of CoQ10. This reaction can be represented as:CoQ10redred + LOOH → CoQ10oxox + LOH (Doll et al., 2019). The reduction in ubiquinol diminishes the cell’s capacity to neutralize LPO, exacerbating oxidative damage.

#### 2.3.3 Impaired regeneration of ubiquinol

CoQ10 reductases in the body use NADH or NADPH as electron donors to reduce ubiquinone back to ubiquinol (Cadenas et al., 2022). Studies have shown that certain mitochondrial enzymes, including complexes I and II of the respiratory chain, transfer electrons to ubiquinone, regenerating it to its reduced form. Similar mechanisms may also occur in other electron transport systems, such as the endoplasmic reticulum (Martínez-Reyes et al., 2020).

When these antioxidant systems are imbalanced, susceptibility to ferroptosis increases significantly. Furthermore, these systems often interact (Fang et al., 2019). For example, GPX4, a key regulator of ferroptosis, relies on GSH to detoxify LPO. When the CoQ10 system is impaired, GPX4’s dependence on GSH intensifies, further depleting cellular GSH reserves. Excessive oxidative stress, coupled with dysfunction of antioxidant systems, leads to mitochondrial ROS accumulation (Dixon et al., 2012), exacerbating oxidative stress and creating a vicious cycle that heightens the risk of ferroptosis (Bedard and Krause, 2007).

## 3 Ferroptosis and BC

BC can be treated through various methods, including surgery, chemotherapy, and radiotherapy. However, reducing the incidence and mortality of BC remains a significant challenge (Barzaman et al., 2020). Studies have demonstrated that BC exhibits a heterogeneous phenotype in terms of ferroptosis-related metabolites and metabolic pathways, characterized by oxidized phosphatidylethanolamine and altered GSH metabolism (Yang F. et al., 2023). Importantly, BC has been identified as a lipid- and iron-rich tumor (Martinez-Outschoorn et al., 2017; Xiao et al., 2022), making ferroptosis induction a promising therapeutic strategy. This mechanism primarily disrupts cancer cell metabolism, inducing cell death through redox imbalance and increased intracellular ROS levels (Tang et al., 2021) (Mou et al., 2019). Furthermore, research has shown that targeting the ferroptosis pathway in BC may enhance therapeutic sensitivity (Yang F. et al., 2023). Conversely, inhibiting this pathway can increase resistance to other chemotherapeutic agents (Xu et al., 2023).

Studies have shown that mutations in the tumor suppressor genes BRCA1 and BRCA2 increase the risk of BC (Liu L. et al., 2020). The loss of function in these genes impairs DNA repair, thereby promoting tumorigenesis (Ben Ayed-Guerfali et al., 2021). Similarly, deletion of the tumor suppressor gene PTEN, which normally inhibits cellular proliferation, invasion, and metastasis, accelerates tumor progression (Chen et al., 2022a). Mutations in other genes, such as CHEK2, ATM, PALB2, BRIP1, and CDH1, allow cancer cells to evade immune surveillance and develop resistance to conventional therapies (Neves et al., 2023; Hansen et al., 2015; Piccolo et al., 2014). In response, researchers have focused on discovering novel anticancer compounds, leading to the identification of ferroptosis inducers (Dolma et al., 2003; Yagoda et al., 2007). Notably, cancer cells resistant to standard treatments have shown increased sensitivity to ferroptosis inducers (Viswanathan et al., 2017; Hangauer et al., 2017; Tsoi et al., 2018).

The development of ferroptosis-based therapies for BC remains in its early stages. Current research focuses on several non-targeted approaches aimed at promoting ferroptosis by enhancing cellular uptake of iron, peroxides, and other substances to eliminate BC cells. Concurrently, efforts are being made to develop targeted therapies that modulate ferroptosis-related molecules, such as enzymes (Hassannia et al., 2019; Hassannia et al., 2018). For example, silencing the GPX4 gene using RNA interference or CRISPR/Cas9, or inhibiting GPX4 activity with ferroptosis inducers like RSL3, can induce ferroptosis by reducing GPX4 levels (Yang et al., 2014; Yang et al., 2016). Targeting GSH metabolism, such as by inhibiting interferon-gamma (IFN-γ), can suppress the XC- system, reducing cystine uptake and GSH synthesis (Sato et al., 1995; Koppula et al., 2021). Similarly, inhibiting glutathione reductase or decreasing NADPH supply can hinder GSH reduction, thereby promoting ferroptosis (Niu et al., 2021). Additionally, silencing FSP1 decreases the production of ferroptosis suppressor proteins, reducing levels of reduced coenzyme Q10 and further potentiating ferroptosis (Wang H. et al., 2021). Iron metabolism plays a central role in ferroptosis, with transferrin (TF) and transferrin receptor 1 (TFR1) regulating iron uptake. Overexpression of TF and TFR1, or inhibition of the iron export pump FPN, increases intracellular iron concentration and LPO, thereby promoting ferroptosis (Bogdan et al., 2016; Gammella et al., 2017; Geng et al., 2018; Torti and Torti, 2020). Finally, certain immune cells, such as CD8^+^ T cells, can enhance the sensitivity of BC cells to ferroptosis (Wang W. et al., 2019). Ferroptosis can also enhance the immune function of cells like neutrophils, aiding BC treatment (Yotsumoto et al., 2017). By altering the tumor microenvironment, ferroptosis may influence tumor prognosis and suppress tumor cell proliferation (Kim et al., 2023).

## 4 The role of ncRNA in BC

### 4.1 miRNA and BC

miRNAs are short RNA molecules derived from longer stem-loop precursors that bind to and inhibit messenger RNA (mRNA) (Chen, 2016). miRNAs exert their function by sequence-specific binding to target RNAs, thereby repressing gene expression (Bartel, 2009; Lee et al., 2009). This process is not solely governed by direct RNA interactions but also involves effector proteins within the miRISC complex (Bartel, 2009). Beyond their role in post-transcriptional gene regulation, miRNAs can recruit their respective ribonucleoprotein complexes to modulate target translation (Vasudevan et al., 2007). miRNAs regulate numerous biological processes, including stress responses, cell adhesion, motility, inflammation (Hata and Kashima, 2016), differentiation, proliferation, senescence, apoptosis, and hematopoiesis (Jensen et al., 2013)—all of which are closely associated with tumorigenesis.

**FIGURE 2:**
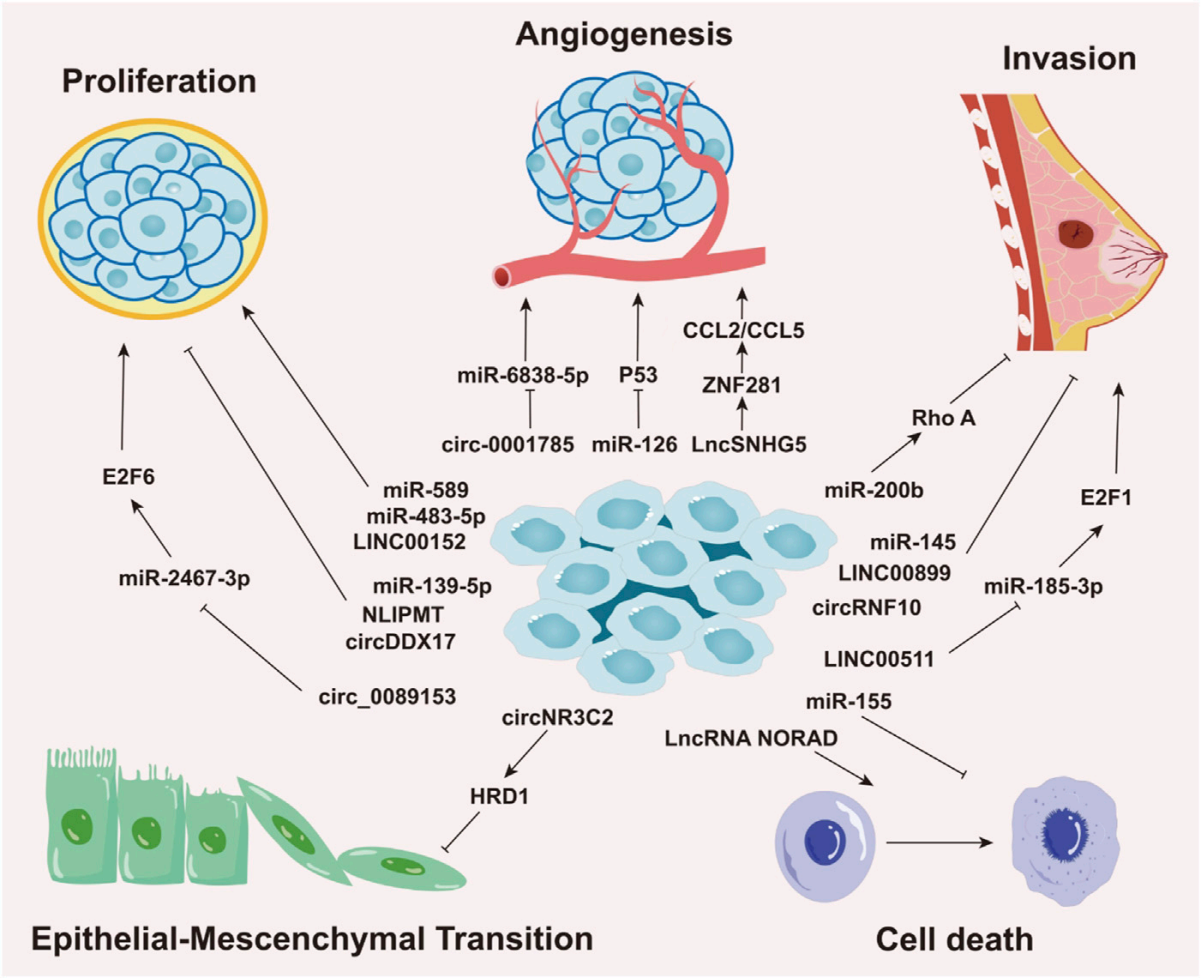
Roles of Non-Coding RNAs in Various Biological Processes of Breast Cancer. This image delineates the roles of different types of non-coding RNAs (ncRNAs), including miRNAs, circRNAs, and lncRNAs, in regulating distinct biological processes in breast cancer, such as cellular proliferation, angiogenesis, invasion, epithelial-mesenchymal transition (EMT), and cell death. The roles and mechanisms of these non-coding RNAs in various biological processes of breast cancer are highly intricate, involving complex regulatory networks that drive cancer progression.

Aberrant miRNA expression is common across cancers and is regulated by epigenetic and tissue-specific mechanisms. This dysregulation is often linked to DNA methylation-mediated gene silencing and distinct cancer phenotypes (Sengupta et al., 2021). Growing evidence highlights the importance of miRNAs as crucial biomarkers in the initiation, progression, detection, and prognosis of BC. For example, in triple-negative breast cancer (TNBC), the most aggressive molecular subtype, miR-200b suppresses metastasis by targeting Rho GTPase-activating protein 18 (ARHGAP18) and promoting RhoA activation (Humphries et al., 2017), underscoring the critical role of miR-200b in BC progression. Elevated levels of miR-10b, miR-34a, and miR-155 are found in patients with metastatic BC (Roth et al., 2010), with circulating miR-10b and miR-373 significantly increased in lymph node-positive patients compared to those without nodal metastasis or healthy controls (Schwarzenbach et al., 2012). Notably, miR-10b has emerged as a potential biomarker for brain (Ahmad et al., 2014)and bone metastasis (Zhao et al., 2012), suggesting its utility for early detection and prognosis. Furthermore, in hormone receptor-positive BC cells resistant to endocrine therapy (tamoxifen), the miR-221/222 cluster is upregulated through the negative regulation of cyclin-dependent kinase inhibitor 1B (p27Kip1) (Hanna et al., 2012; Gan et al., 2014; Miller et al., 2008). p27Kip1, an enzyme inhibitor encoded by the CDKN1B gene, plays a pivotal role in regulating the human cell cycle (Kurozumi et al., 2015; Toyoshima and Hunter, 1994). Therefore, the miR-221/222 cluster has been proposed as a potential therapeutic target and predictor of tamoxifen resistance in BC treatment (Figure 2) (Table 1).

**TABLE 1:** ncRNAs functioning in the process of BC.

ncRNA	Target	Functions in BC	Reference
miR-2467-3p	E2F6	Proliferation (−) Migration (−) Invasion (−) EMT (−)	Gao et al. (2022)
miR-223-3p	FBXW7	Invasion (+) Metastasis (+)	Wang Y. et al. (2022)
miR-382	Peroxisome proliferator-activated receptor γ coactivator-1α (PGC-1α)	Invasion (−) Migration (−) EMT (−)	Zhou et al. (2022a)
miR-379-5p	KIF4A	Proliferation (−) Migration (−) Invasion (−)	Yang et al. (2022)
miR-142-5p	DNA methyltransfer-ase 1 (DNMT1)	Proliferation (−) Migration (−)	Li et al. (2022b)
miR-556-5p	Parathyroid hormone related protein (PTHrP)	Invasion (−) Migration (−) EMT (−)	Zhou et al. (2022b)
miR-181a-5p	N-myc downstream-regulated gene (NDRG) 2	Proliferation (+) Invasion (+) Glycolysis (+)	Zhai et al. (2022)
miR-409	AT-rich sequence-binding protein 1 (SATB1)	Proliferation (−) Invasion (−)	Chen et al. (2022c)
miR-183	SIN3A (SWI-independent-3) chromatin modification complexes	Migration (+) Invasion (+) Metastasis (+)	Davenport et al. (2022)
miR-606	Stanniocalcin 1 (STC1)	Proliferation (−) Stem cell-like ability (−) Migration (−) Invasion (−)	Choi et al. (2024)
miR-148b-3p	TSC2/mTORC1 signaling pathway	Proliferation (+) Migration (+) Invasion (+)	Hao et al. (2023)
miR-7-5p	receptor-like tyrosine kinase (*RYK*)	Migration (+) Invasion (+) EMT (+)	Liang et al. (2022)
miR-181c	MAP4K4	Cells apoptosis (+) Proliferation (−) Migration (−)	Xie et al. (2022c)
miR-219-5p	TBXT	Migration (−) Invasion (−) EMT (−)	Ye et al. (2021)
miR-146a	NM23-H1 gene	Proliferation (+) Migration (+) Invasion (+)	Chen et al. (2020)
miR-150-5p	MYB	Proliferation (−)	Jia et al. (2021)
miR-21	Leucine zipper transcription factor-like 1 (LZTFL1)	Proliferation (−) Migration (−)	Wang H. et al., 2019
miR-589-5p	Histone deacetylase 3 (HDAC3)	Proliferation (−)	Rahbari et al. (2022)
miR-138-5p	rhomboid domain-containing protein 1 (RHBDD1)	Migration (−) Invasion (−) EMT (−)	Zhao et al. (2019)
miRNA-874-3p	Voltage-dependent anion channel 1 (VDAC1)	Migration (−) Invasion (−) Proliferation (−)	Yang H. et al. (2023)
LncRNA NORAD	pro-metastatic protein S100P	Migration (−) Invasion (−)	Tan et al. (2019)
NAT PDCD4-AS1 lncRNA	*PDCD4*	Migration (−) Proliferation (−)	Jadaliha et al. (2018)
*LncKLHDC7B*	KLHDC7B gene	Apoptosis (+) Migration (−) Invasion (−)	Beltrán-Anaya et al. (2019)
lncRNA GAS5	miR-378a-5p/SUFU signaling	Apoptosis (+)	Zheng et al. (2020a)
lncRNA MT1JP	miRNA-214/RUNX3 Axis	Invasion (−) Migration (−) Proliferation (−) Apoptosis (+)	Ouyang et al. (2020)
LncRNA NEF	miRNA-155	Migration (−) Invasion (−)	Song et al. (2019)
LncRNA XIST	IL-6/STAT3 signaling	Proliferation (+) Migration (+) Cancer stemness (+)	Ma et al. (2023)
LncRNA MEG3	miR-141-3p/RBMS3 axis	Proliferation (−) Apoptosis (+)	Dong et al. (2021)
lncRNA NLIPMT	Glycogen synthase kinase 3β (GSK3β)	Proliferation (−) Migration (−) Invasion (−) Metastasis (−)	Jiang et al. (2019)
LncRNA GAS5	miR-196a-5p	Proliferation (−) Invasion (−)	Li et al. (2018)
lncRNA DANCR	miR-4319/VAPB axis	Proliferation (+) Metastasis (+)	Jia et al. (2022)
LncRNA H19	p53/TNFAIP8 pathway	Invasion (+) Metastasis (+)	Li Y. et al. (2020)
LncRNA NEAT1	miR-133b/CXCL12 axis	Paclitaxel resistance (+) Migration (+)	Wei et al. (2023)
*HOTAIR*	*HOTAIR*/*miR-203*/*CAV1* axis	Proliferation (+) Invasion (+) Migration (+)	Shi et al. (2022)
LINC00152	LINC00152-KLF5 loop	Proliferation (+)	Li et al. (2021)
lncRNA FEZF1-AS1	miR-30a/Nanog axis	Proliferation (+) Invasion (+) Migration (+)	Zhang et al. (2018b)
LINC00461	miR-144-3p/KPNA2 axis	Migration (+) Invasion (+)	Zhang et al. (2020c)
*NEAT1*	PGK1/PGAM1/ENO1 multienzyme complex	Glycolysis (+) Mor growth(+)	Park et al. (2021)
LINC01857	miR-2052/CENPQ axis	Metastasis (+) Vascularization (+) Migration (+)	Qian et al. (2022)
LINC00511	LINC00511/miR-150/MMP13 axis	Proliferation (+) Migration (+) Invasion (+)	Shi et al. (2021)
circ-UBR1	miR-1299/CCND1 axis	Proliferation (+) Metastasis (+) Apoptosis (−)	Zhang et al. (2021)
circDDX17	miR-605	Proliferation (−) Apoptosis (+)	Peng and Wen (2020)
circ_103809	PI3K/AKT signaling	Cell apoptosis (−) Proliferation (+)	Qiu et al. (2020)
circ_0000526	miR-492/SOCS2 axis	Proliferation (−) Metastasis (−) Apoptosis (+)	Wang W. B et al., 2021
circSEPT9	circSEPT9/miR-637/LIF axis	Proliferation (+) Migration (+) Invasion (+)	Zheng et al. (2020b)
circEPSTI1	miR-145/ERBB3 axis	Proliferation (+) Migration (+) Invasion (+)	Zhang et al. (2022b)
circGFRA1	circGFRA1-miR-1228-AIFM2 axis	Infiltration (+) Proliferation (+) Migration (+)	Bazhabayi et al. (2021)
circKIF4A	miR-152/ZEB1 axis	Migration (+) Invasion (+) Apoptosis (−)	Jin et al. (2020)
CircUBE2D2	miR-512-3p/CDCA3 axis	Proliferation (+) Migration (+) Invasion (+)	Dou et al. (2020)
circHIF1A	NFIB	Growth (+) Metastasis (+)	Chen et al. (2021c)
circNR3C2	circNR3C2/miR-513a-3p/HRD1/Vimentin axis	Proliferation (−) Migration (−) Invasion (−) EMT (−)	Fan et al. (2021)
circANKS1B	miR-148a/152-3p	Invasion (+) Metastasis (+)	Zeng et al. (2018)
circHIPK3	miR-326	Proliferation (+) Migration (+) Invasion (+)	Qi et al. (2021)
ciRS-7	miR-1299	Migration (+) Invasion (+)	Sang et al. (2018)
hsa_circ_0025202	miR-182-5p/FOXO3a axis	Proliferation (−) Colony formation (−) Migration (−)	Sang et al. (2019)
circ_0045881	miR-214-3p	Invasion (−) Migration (−)	Ren et al. (2024)
circRNF10	DHX15	Proliferation (−) Migration (−)	Zheng et al. (2023b)
Circ-FOXO3	WHSC1-H3K36me2-Zeb2 axis	Proliferation (−) Migration (−) Metastasis (−)	Chen et al. (2024)
circNFIB	Inhibits synthesis of AA by regulating phospholipase	Invasion (−) Metastasis (−)	Zhong et al. (2024)
circ_ATAD3B	miR-570-3p/MX2 pathway	Proliferation (−)	Song et al. (2023b)

+means promoting, − means inhibiting.

### 4.2 lncRNA and BC

lncRNAs are transcripts longer than 200 nucleotides that do not encode proteins (Mattick and Rinn, 2015). They regulate gene expression through epigenetic modifications, as well as transcriptional and post-transcriptional mechanisms. lncRNAs engage in base-pairing with other RNA molecules (e.g., mRNA, miRNA, or DNA), facilitating direct interactions at the primary structure level (Novikova et al., 2013). At the secondary structure level, lncRNAs perform their roles through base-pairing or ribonucleotide backbone interactions (Cruz and Westhof, 2009; Mercer and Mattick, 2013). In addition to protein interactions mediated by their spatial conformation, lncRNAs recognize other nucleic acids through base-pairing, guiding proteins to specific loci and thereby broadening their functional roles in cancer.

The relationship between lncRNAs and BC has attracted increasing attention due to their critical role in regulating various cancer-related processes, including cell proliferation, invasion, migration, apoptosis, epithelial-mesenchymal transition (EMT), and drug resistance across multiple malignancies (Jin et al., 2021; Wang CJ. et al., 2019; Guo et al., 2019). In BC, lncRNAs influence prognosis through several mechanisms, which include: (Makhoul et al., 2018): Peptide-Encoding lncRNAs: Recent studies have revealed that certain lncRNAs harbor hidden open reading frames capable of encoding functional peptides. For example, LINC00908 encodes a peptide, ASRPS, which exerts antitumor effects by inhibiting angiogenesis in TNBC (Wang et al., 2020). (Nik-Zainal et al., 2016) mRNA Stability Modulation: lncRNAs also regulate mRNA stability in BC cells. For instance, the PDCD4-AS1 lncRNA stabilizes the mRNA of the tumor suppressor gene PDCD4 by forming double-stranded RNA, which interacts with RNA decay factors such as HuR, thereby inhibiting BC cell proliferation and migration (Jadaliha et al., 2018). (Duffy et al., 2012) lncRNAs in Intercellular Communication: Emerging studies suggest that certain lncRNAs can be encapsulated in extracellular vesicles (EVs) and mediate intercellular communication. For instance, EVs released by cancer-associated fibroblasts (CAFs) containing the lncRNA SNHG3 are absorbed by BC cells, where SNHG3 acts as a sponge for miR-330-5p, thereby reprogramming cellular metabolic pathways (Jin et al., 2021). Overall, lncRNAs participate in multiple mechanisms that drive BC development and progression. Research suggests that lncRNAs hold significant potential as molecular biomarkers for BC, offering both research and clinical value (Yuan et al., 2021).

Through these mechanisms, lncRNAs exhibit oncogenic and tumor-promoting activities, influencing cancer development by engaging in or disrupting key cellular pathways (Zeng et al., 2022) (Figure 2) (Table 1).

### 4.3 circRNA and BC

circRNAs are covalently closed RNA molecules formed through the back-splicing of exons or introns (Jeck et al., 2013). circRNAs are involved in a range of biological processes. Due to their miRNA-binding sites, circRNAs can act as miRNA sponges, modulating miRNA expression (Panda, 2018). In addition to regulating transcription (Stoll et al., 2020), circRNAs may also be translated into proteins (Yang et al., 2017), influence protein function by shuttling between the nucleus and cytoplasm, and promote cellular senescence (Fang et al., 2018). Furthermore, circRNAs serve as protein scaffolds, facilitating protein-protein interactions (Du et al., 2017).

The mechanisms through which circRNAs influence BC are diverse (Wang Z. et al., 2023): (Makhoul et al., 2018) circRNAs as miRNA Sponges: circRNAs can modulate gene expression by sequestering miRNAs, either upregulating or downregulating their targets. For example, circ_0089153 sponges miR-2467-3p, alleviating its inhibitory effect on E2F6, thus promoting BC cell proliferation, migration, invasion, and EMT (Gao et al., 2022). (Nik-Zainal et al., 2016) circRNAs as RNA-Binding Protein (RBP) Complexes: circRNAs can interact with RBPs involved in pre-mRNA transcription, splicing, polyadenylation, and RNA degradation. The RBP human antigen R (HuR) stabilizes mRNA and regulates its processing (Lebedeva et al., 2011). Experimental evidence shows that circ-1073 binds to HuR in BC cells, increasing the levels of caspases 3/9 and E-cadherin, thereby inhibiting oncogenic activity (Yi et al., 2020). (Duffy et al., 2012) circRNAs as Translation Templates: A growing area of research suggests that circRNAs may also function as templates for translation (Zhang M. et al., 2018). For example, circFAM53B encodes a unique peptide in BC cells that binds to HLA-I molecules, enhancing antitumor immunity by inducing higher levels of immunoactive substances such as IFNγ and perforin (Huang et al., 2024). Additionally, circCAPG encodes a peptide known as CAPG-71AA, which promotes tumor growth in BC (Song R. et al., 2023). Numerous circRNAs have been identified as key regulators in BC pathogenesis, with potential applications in diagnosis, prognosis, and therapeutic interventions.

In summary, circRNAs play a central role in regulating tumor proliferation and the surrounding microenvironment, positioning them as crucial mediators in the initiation and progression of BC (Figure 2) (Table 1).

## 5 The relationship between ncRNA and ferroptosis

### 5.1 The relationship between miRNAs and ferroptosis

**FIGURE 3:**
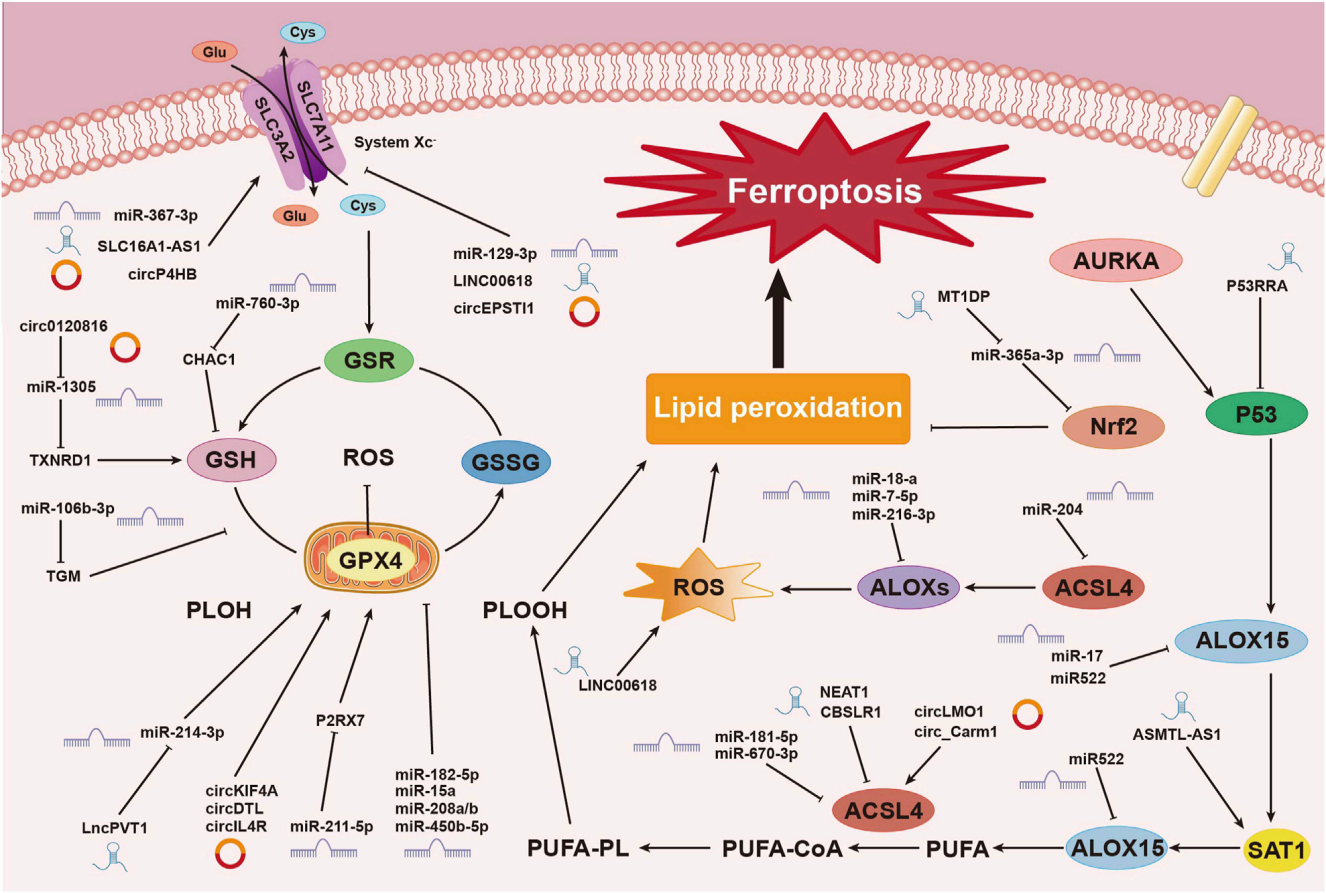
Mechanisms of Non-Coding RNAs in Regulating Ferroptosis. This image illustrates the mechanisms by which non-coding RNAs (ncRNAs), including miRNAs, circRNAs, and lncRNAs, regulate the process of ferroptosis, a form of cell death driven by iron-dependent lipid peroxidation. ncRNAs play pivotal roles at various stages of this process. Non-coding RNAs influence the process of ferroptosis in breast cancer cells through multiple pathways and mechanisms. These include regulating system Xc⁻, modulating glutathione metabolism and redox reactions, controlling lipid peroxidation, and influencing ROS production. These complex interactions and regulatory networks involving miRNAs, circRNAs, and lncRNAs underscore the critical role of ncRNAs in ferroptosis within the context of breast cancer.

Research has shown that various miRNAs regulate iron metabolism by modulating intracellular iron levels, thereby influencing ferroptosis (Zuo et al., 2022). For example, miR-545 binds to TF mRNA, inhibiting its expression (Zheng et al., 2021), while miR-214 and miR-367-3p target and suppress TFRC expression, reducing iron absorption and preventing ferroptosis (Lu et al., 2020; Huang J. et al., 2023). Additionally, miR-7-5p downregulates mitochondrial TF, lowering Fe^2^⁺ levels and inhibiting ferroptosis (Tomita et al., 2019). In the context of ulcerative colitis (UC), miR-375-3p binds to the transmembrane iron transporter SLC11A2, downregulating its transcription and blocking iron absorption and metabolism, thus preventing ferroptosis (Luo and Chen, 2023). MiR-19a suppresses ferroptosis in colorectal cancer by modulating iron metabolism and inhibiting the ferroptosis-inducing factor iron-responsive element-binding protein 2 (IREB2) (Fan et al., 2022). Conversely, miR-761 promotes ferroptosis by reducing hepcidin levels, preventing the degradation of ferroportin 1 (FPN1) (Zheng R. et al., 2023). MiR-30b-5p suppresses Pax3 to downregulate FPN1 transcription, inducing ferroptosis in trophoblast cells, which is associated with preeclampsia (Zhang H. et al., 2020). MiR-19b-3p directly targets and reduces ferritin heavy chain 1 (FTH1) expression, resulting in increased free iron and promoting ferroptosis in lung cancer (Zhang R. et al., 2022). Furthermore, miR-129-5p targets and downregulates PROM2, inhibiting iron efflux and enhancing ferroptosis in non-small cell lung cancer (NSCLC) (Luo W. et al., 2021) (Figure 3).

### 5.2 The relationship between lncRNAs and ferroptosis

LINC00618 has been shown to enhance ferroptosis by increasing levels of ROS and iron, both of which are hallmark indicators of ferroptosis, while also downregulating SLC7A11 expression. Deletion of LINC00618 significantly reduces ROS and iron levels in cancer cells, highlighting its role in enhancing sensitivity to ferroptosis through classical pathways (Wang Z. et al., 2021). In contrast, lncPVT1 suppresses ferroptosis by downregulating miR-214-3p, which leads to increased GPX4 expression and subsequently promotes cancer progression (He et al., 2021).

In summary, lncRNAs exert multilayered regulatory control over ferroptosis in cancer cells. As demonstrated, the mechanisms through which lncRNAs influence ferroptosis are complex and interconnected. Although categorizing these mechanisms may be challenging, their roles are of significant importance. This complexity emphasizes the need for further exploration, making this area a key focus in current cancer research (Figure 3).

### 5.3 The relationship between circRNAs and ferroptosis

circLMO1 and circ_Carm1 promote ferroptosis by upregulating ACSL4 expression (Mao and Liu, 2022). In contrast, circKIF4A (Chen W. et al., 2021), circDTL (Shanshan et al., 2021), cmmu_circRNA_000030 (Jin et al., 2022), and circIL4R (Xu et al., 2020) inhibit ferroptosis by upregulating GPX4 expression. Similarly, circ_0067934 (Wang HH. et al., 2021), and circP4HB (Pan et al., 2022) upregulate SLC7A11 expression, affecting the system Xc− and inhibiting ferroptosis. CircGFRA1 (Bazhabayi et al., 2021) upregulates AIFM2, which encodes FSP1, further suppressing ferroptosis. Recent research has identified numerous circRNAs that regulate ferroptosis through classical pathways, with ongoing studies continuing to enhance our understanding of these mechanisms (Figure 3).

## 6 Ferroptosis-related ncRNAs and their association with BC

### 6.1 Ferroptosis-related miRNAs and their association with BC

Ferroptosis has become a central focus in BC research. Studies utilizing 3′UTR luciferase assays have demonstrated that miR-5096 targets SLC7A11, thereby promoting ferroptosis in BC cells by downregulating SLC7A11 expression. This mechanism increases ROS production, iron accumulation, and lipid peroxidation, while inhibiting BC cell proliferation, colony formation, migration, and invasion. Notably, miR-5096 induces ferroptosis more effectively in TNBC cells compared to other BC subtypes. Furthermore, miR-5096 reduces the metastatic potential of MDA-MB-231 cells in a zebrafish larvae xenotransplantation model. These findings suggest that miR-5096 may serve as a promising therapeutic target in BC, particularly in TNBC (Yadav et al., 2021).

MiR-128-3p, which is downregulated in BC patients, plays a critical role in various biological processes. By directly targeting SP1 mRNA, miR-128-3p inhibits TGF-β1-induced migration, invasion, and cell cycle progression (Nalla et al., 2022). CD98 heavy chain (CD98hc), a type II transmembrane glycoprotein, interacts with several light chain amino acid transporters, including xCT, LAT1, and y + LAT2, and is involved in the intersection of oxidative stress and amino acid metabolism (de la Ballina et al., 2016). Upregulation of CD98hc promotes cell proliferation, EMT, adhesion, and polarity (Feral et al., 2005), while its downregulation can trigger ferroptosis. Inhibition of SP1 affects CD98hc expression (Yan et al., 2007). Consequently, studies have shown that empagliflozin (EMPA), functioning as a miR-128-3p mimetic, suppresses SP1 expression, reduces CD98hc levels, sensitizes cells to ferroptosis, and may serve as a potential adjunct in BC chemotherapy (Nalla and Khairnar, 2024).

MiR-335-5p exhibits tumor-suppressive properties in various cancers, including BC (Gao et al., 2021; Qian et al., 2021; Zhang S. et al., 2023). Adenosine deaminase acting on RNA 1 (ADAR1), an RNA editing enzyme that converts adenosine to inosine within double-stranded RNA, plays a crucial role in the initiation and progression of several cancers and is overexpressed in BC (Li Y. et al., 2022). Studies have shown that ADAR1 downregulates miR-335-5p in an editing-independent manner, promoting Sp1 expression, upregulating GPX4 levels, and inhibiting ferroptosis in BC. These findings offer new insights into the role of ADAR1 in BC progression (Yin et al., 2024).

Lidocaine, a commonly used local anesthetic, exhibits antitumor activity under experimental conditions, including the inhibition of BC cell viability suppressed by erastin, highlighting its potential role in BC progression. Lidocaine treatment induces the accumulation of Fe^2^⁺, iron, and ROS in BC cells, thereby inhibiting cell proliferation, while promoting apoptosis and reducing cell invasion and migration (Gao et al., 2018; Khan et al., 2019). Studies have shown that lidocaine enhances ferroptosis by upregulating miR-382-5p, which downregulates SLC7A11 and suppresses malignant BC progression. Inhibition of miR-382-5p blocks lidocaine-induced ferroptosis in BC cells. However, the clinical potential of lidocaine in BC treatment requires further investigation (Sun et al., 2021). Metformin, a widely prescribed oral hypoglycemic agent, has demonstrated inhibitory effects on the proliferation and metastasis of various cancers, including BC (Wang et al., 2017; Mallik and Chowdhury, 2018). Metformin suppresses cell proliferation, upregulates Fe^2^⁺ and ROS levels, inhibits GPX4 expression, and induces ferroptosis in BC cells (Yang et al., 2021). Furthermore, metformin promotes ferroptosis by upregulating miR-324-3p, which directly targets GPX4 for downregulation (Hou et al., 2021). These findings suggest that metformin, in combination with miR-324-3p, holds promise as a novel therapeutic strategy for cancer treatment.

The investigation of ferroptosis-related miRNAs not only provides new opportunities for BC treatment but also enhances our understanding of the mechanisms through which miRNAs regulate ferroptosis. Future research is anticipated to further elucidate their clinical potential in therapeutic applications.

### 6.2 The relationship between ferroptosis-associated lncRNAs and BC

In the context of BC therapies, ferroptosis has emerged as a critical mechanism in tumor treatment. lncRNAs have garnered significant attention due to their multifaceted roles in tumorigenesis and the regulation of ferroptosis. Several lncRNAs have been implicated in the modulation of ferroptosis within BC cells, including AC092916.1, L133467.1, USP30-AS1, AC108474.1, LINC01235, AL365356.1, AC072039.2, AC012213.3, LIPE-AS1, MAPT-AS1, and TDRKHAS1. Notably, lncRNA USP30-AS1 exhibits a significant co-expression pattern with nine ferroptosis-related genes, including SOCS1, CAPG, IFNG, PML, TNFAIP3, NCF2, SLC2A6, GCH1, and CYBB. This co-expression suggests that upregulation of USP30-AS1 may be associated with improved survival in BC patients. Additionally, lncRNA LIPE-AS1 interacts with key ferroptosis-related genes, such as GPX4, PHKG2, EGLN2, MAPK14, and HRAS, offering new insights into potential therapeutic strategies for improving patient prognosis. Similarly, AC108474.1 has been shown to interact with five ferroptosis-related genes, including HIC1, ISCU, PLIN4, CAV1, and TAZ, suggesting its potential role as a protective factor in BC (Xiang et al., 2024). Research has also identified specific lncRNAs that are strongly associated with BC prognosis. LINC01871, LINC02384, LIPE-AS1, and HSD11B1-AS1 have been classified as low-risk ferroptosis-related lncRNAs (FRLncRNAs), while LINC00393, AC121247.2, AC010655.2, LINC01419, PTPRD-AS1, AC099329.2, OTUD6B-AS1, and LINC02266 are considered high-risk FRLncRNAs.

Recent studies have identified LncFASA as a significant regulator in BC. LncFASA interacts with a specific domain of peroxiredoxin PRDX1, promoting its liquid-liquid phase separation, a process that impairs the enzyme’s peroxidase activity and affects the SLC7A11-GPX4 signaling axis, which is crucial for maintaining cellular oxidative stress homeostasis (Lovatt et al., 2020). This disruption leads to the accumulation of lipid ROS and triggers ferroptosis (Neumann et al., 2003). Notably, elevated LncFASA expression is strongly correlated with the formation of PRDX1 droplets and a favorable prognosis in BC patients (Fan et al., 2024).

Conversely, certain lncRNAs suppress ferroptosis and promote BC progression. For example, a novel protein encoded by the lncRNA HCP5, called HCP5-132aa, promotes the growth of TNBC by regulating GPX4 and reducing ROS levels, thereby inhibiting ferroptosis. Kaplan-Meier survival analysis reveals that high HCP5-132aa expression is associated with an increased risk of BC-related mortality, positioning it as a risk factor for TNBC progression (Tong et al., 2023). Additionally, specific lncRNAs, such as RUNX1-IT1, are selectively upregulated during cancer progression. RUNX1-IT1 is significantly elevated in BC tissues and correlates with larger tumor size and more advanced clinical stages. Mechanistically, RUNX1-IT1 binds to the m6A reader protein IGF2BP1, stabilizing GPX4 mRNA and inhibiting ferroptosis (Wang S. et al., 2023). Similarly, the lncRNA DSCAM-AS1 binds to m6A-modified SLC7A11 mRNA, enhancing its stability and inhibiting ferroptosis (Yan et al., 2024).

LINC00460 expression is significantly elevated in BC tissues compared to normal tissues, promoting cancer cell proliferation and inhibiting ferroptosis by sponging miR-320a and upregulating myelin and lymphocyte protein 2 (MAL2) (Zhang C. et al., 2023). MAL2 has been implicated in cancer progression through various mechanisms (Fang et al., 2021; Jeong et al., 2021; López-Coral et al., 2020). Research suggests that MAL2 overexpression can reverse the effects of LINC00460 knockdown on both proliferation and ferroptosis in BC cells (Zhang C. et al., 2023).

Given the specificity of lncRNAs in BC cells, several prognostic models have been developed (Liu Q. et al., 2020). One such model includes nine metabolism-related lncRNAs—SIRLNT, SIAH2-AS1, MIR205HG, USP30-AS1, MIR200CHG, TFAP2A-AS1, AP005131.2, AL031316.1, and C6orf99—which shows potential for improving predictive accuracy and enabling personalized treatment for BC patients. Prospective validation of these lncRNA signatures could further enhance their clinical utility (Ge et al., 2024). Beyond prognostic models, various therapeutic strategies targeting lncRNAs have been explored. For example, metformin has been shown to downregulate lncRNA H19, inducing ferroptosis in BC cells by increasing lipid ROS levels (Alimova et al., 2009; Chen et al., 2022b). Additionally, lncRNAs contribute to overcoming drug resistance, with targeting LINC00152 demonstrated to increase BC sensitivity to tamoxifen by stabilizing PDE4D mRNA. This stabilization raises cytosolic Ca^2^⁺ levels, a key regulator of ferroptosis, thereby enhancing tamoxifen efficacy through the induction of ROS and ferroptosis. (Gomez et al., 2002; Pedrera et al., 2021; Saatci et al., 2023). In conclusion, lncRNAs play critical roles in BC development and ferroptosis, presenting promising opportunities for future research. Their involvement in tumor therapy, staging, and prognosis suggests they could serve as valuable therapeutic targets.

### 6.3 The relationship between ferroptosis-associated circRNAs and BC

Although research on ferroptosis-related circRNAs in BC cells remains limited, we have compiled the existing findings to provide a basis for future exploration.

CircGFRA1 has been shown to promote the malignant progression of HER-2 positive BC by acting as a sponge for miR-1228 and enhancing the expression of AIFM2, an essential NADH oxidase. CircGFRA1 is upregulated in HER-2 positive BC cells and tissues, positioning it as a potential biomarker for BC diagnosis. Silencing circGFRA1 inhibits the proliferation of HER-2 positive BC cells and attenuates invasion and metastasis, highlighting its therapeutic potential (Bazhabayi et al., 2021). The mechanisms underlying this process are as follows: (Makhoul et al., 2018) circGFRA1 sponges miR-1228, leading to the upregulation of AIFM2, which inhibits ferroptosis mediated by ubiquinone and thus promotes BC progression (Bazhabayi et al., 2021; Chen and Xie, 2020); (Nik-Zainal et al., 2016) in experimental models, silencing circGFRA1 in cancer cells reduces the GSH/GSSG ratio, depletes GPX4, accumulates highly toxic lipid ROS, and induces ferroptosis (Seibt et al., 2019). Therefore, circGFRA1 modulates ferroptosis through multiple pathways in BC cells.

Circ_0000643 regulates ferroptosis in BC cells through the FOXQ1/circ_0000643/miR-153/SLC7A11 axis. FOXQ1, a member of the Forkhead box protein family, is an oncogenic transcription factor highly expressed in various tumors and associated with poor prognosis (Dong et al., 2022). SLC7A11, a downstream target of miR-153, is regulated by circ_0000643, which acts as a sponge for miR-153 in BC cells. By upregulating SLC7A11, circ_0000643 promotes BC progression and inhibits ferroptosis (Lin et al., 2020). Furthermore, FOXQ1 enhances the expression of circ_0000643 in BC by binding to the promoter region of ZFAND6, establishing circ_0000643 as a critical component in this regulatory axis (Huang X. et al., 2023).

Emerging studies indicate that circRNAs modulate ferroptosis and influence drug resistance in BC cells. For example, circ-BGN directly interacts with the deubiquitinase OTUB1 and the ferroportin-related protein SLC7A11, enhancing OTUB1-mediated deubiquitination of SLC7A11, which inhibits ferroptosis. Notably, the small-molecule ferroptosis inducer Erastin significantly reduces tumor volume in trastuzumab-resistant BC cells, with increased efficacy when circ-BGN is co-silenced. This suggests that Erastin may restore the antitumor effects of trastuzumab by inducing ferroptosis (Wang S. et al., 2022). Furthermore, SRSF1, circSEPT9, and GCH1 are upregulated in triple-negative BC (TNBC) cells. Downregulation of SRSF1 reduces the IC50 of cisplatin (DDP) in both parental and resistant TNBC cells, inhibiting cell viability and proliferation, decreasing GSH/SLC7A11 levels, and increasing Fe3+/ROS/MDA/ACSL4 levels, thereby promoting ferroptosis. SRSF1 binds to circSEPT9, which in turn upregulates GCH1 by preventing its ubiquitination, thus enhancing GCH1 protein levels. Overexpression of circSEPT9 and GCH1 suppresses ferroptosis, ultimately reducing the chemosensitivity of TNBC cells to DDP (Song et al., 2024).

In conclusion, while research in this field remains in its early stages, existing studies provide valuable insights into the diagnosis and treatment of BC, highlighting its significant potential. Further investigations are needed to explore additional therapeutic strategies, validate current approaches, and translate promising diagnostic and therapeutic targets into clinical practice (Table 2).

**TABLE 2:** Ferroptosis-related ncRNAs and Their Association with BC.

ncRNA	Mechanism	Function	Reference
miR-5096	Target SLC7A11	Ferroptosis (+) and BC(−)	Yadav et al. (2021)
miR-499a-5p	Targe TMEM189	Ferroptosis (+) and BC(−)	Fan et al. (2023)
miR-128-3p	Target SP1 mRNA/CD98hc	Ferroptosis (+) and BC(−)	de la Ballina et al. (2016), Feral et al. (2005), Yan et al. (2007)
miR-335-5p	Target Sp1/GPX4	Ferroptosis (+) and BC(−)	Yin et al. (2024)
miR-382-5p	Downregulate SLC7A11	Ferroptosis (+) and BC(−)	Sun et al. (2021)
miR-324-3p	Target GPX4	Ferroptosis (+) and BC(−)	Hou et al. (2021)
LINC01871	IFNG co-expressed	Ferroptosis (+) and BC(−)	Xu et al. (2021), Mathias et al. (2021)
lncRNA P53RRA	Interact with G3BP	Ferroptosis (+) and BC(−)	Mao et al. (2018)
LncFASA	Upregulate the formation of PRDX1	Ferroptosis (+) and BC(−)	Fan et al. (2024)
lncRNA HCP5	Target Xc-/GSH/GPX4	Ferroptosis (−) and TNBC(+)	Tong et al. (2023)
LINC00460	Target miR-320a/MAL2	Ferroptosis (−) and BC(+)	Zhang et al. (2023b)
LINC00152	Target PDE4D/cAMP/Ca2+	Ferroptosis (−) and BC(+)	Pedrera et al. (2021), Saatci et al. (2023)
lncRNA DSCAM-AS1	Upregulate SLC7A11	Ferroptosis (−) and BC(+)	Yan et al. (2024)
LncRNA RUNX1-IT1 LncRNA H19 CircGFRA1	Upregulate GPX4 promote autophagy Target miR-1228/AIFM2	Ferroptosis (−) and BC(+) Ferroptosis (−) and BC(+) Ferroptosis (−) and BC(+)	Wang H. H. et al. (2023b) Chen et al. (2022b) Bazhabayi et al. (2021)
CircRHOT1 circ-BGN	Target miR-106a-5p/STAT3 Enhancing OTUB1-mediated SLC7A11	Ferroptosis (−) and BC(+) Ferroptosis (−) and HER2-positive BC(+)	Banerjee and Resat (2016) Wang S. et al. (2022)
circ_0000643	Target FOXQ1/circ_0000643/miR-153/SLC7A11	Ferroptosis (−) and BC(+)	Huang X. et al. (2023)
SRSF1	Downregulate GSH/SLC7A11 Upregulate Fe3+/ROS/MDA/ACSL4	Ferroptosis (+) and BC(−)	Song et al. (2024)
circSEPT9	Upregulate GCH1	Ferroptosis (−) and TNBC(+)	Song et al. (2024)

+means promoting, − means inhibiting.

## 7 Conclusion

BC remains the leading cause of cancer-related mortality among women worldwide. Identifying effective treatment strategies and improving patient recovery rates are of critical importance. Recent studies have highlighted the pivotal role of ferroptosis in tumor development, with increased ferroptosis in cells shown to inhibit tumor progression (Bedoui et al., 2020; Shen et al., 2018). Furthermore, ncRNAs, including miRNAs, lncRNAs, and circRNAs, have been implicated in ferroptosis-related biological processes, influencing cancer growth (Chen Li, 2020).

Based on the treatment mechanisms, targeting ncRNAs (ncRNAs) plays a crucial regulatory role in cancer progression and may emerge as a novel therapeutic strategy for combating BC in the future. There are generally two main approaches for targeting ncRNAs: the first involves inhibiting the expression of overexpressed ncRNAs that act on oncogenes, thereby suppressing tumor progression. The second approach aims to activate or upregulate ncRNAs that express tumor suppressor genes to inhibit cancer development.

Despite the potential of ncRNA-based ferroptosis therapy, several challenges remain. The regulatory network of ferroptosis-related ncRNAs in cancer treatment and diagnosis is not yet fully understood. Current therapies targeting tumorigenesis via ncRNA-mediated ferroptosis have limited efficacy, and individual variability in ncRNA expression, along with differing responses to treatment, significantly affects predictability. Further research is required to balance the promotion of ferroptosis for tumor inhibition with the prevention of chemotherapy resistance through ncRNA regulation. While Erastin is a potent ferroptosis inducer, its low solubility and rapid metabolic degradation remain significant drawbacks (Larraufie et al., 2015). In terms of diagnosis, the lack of standardized reference genes for circulating lncRNAs impedes the development of reliable diagnostic methods for BC. Moreover, circulating lncRNAs often exhibit low expression levels, making detection difficult and reducing diagnostic accuracy (Schlosser et al., 2016). Additionally, the anti-breast cancer mechanisms of traditional herbal medicine remain poorly understood, and only a limited number of lncRNA functions have been elucidated. Identifying potential therapeutic targets for lncRNAs is crucial for developing more effective treatments. Identifying potential therapeutic targets for lncRNAs is crucial for developing more effective treatments (Li et al., 2023). The impact of immune cell subsets and signaling pathways on immune checkpoint inhibitor (ICI) treatment responses requires further investigation. Additionally, the relationship between genetic characteristics, immune subtypes, and specific mutations, and how these factors influence treatment outcomes or resistance, needs to be explored. In addition, further development of ICIs and targeted therapy, chemotherapy, radiotherapy, or other immunotherapies (such as new ICIs and chimeric antigen receptor T cells) is needed to more accurately select appropriate combination therapies (Xie Q. et al., 2022). Overall, the clinical application of ncRNA regulation in BC ferroptosis faces significant challenges, including the lack of effective therapeutic agents and unresolved chemotherapy resistance. The unclear targets of ncRNA regulation in ferroptosis will be a major hurdle for clinical implementation.

To address this issue, further investigation is needed into the encapsulation of ferroptosis-inducing agents within protective delivery systems, such as nanoparticles (Larraufie et al., 2015). Additionally, to explore the clinical potential of targeting ferroptosis-related ncRNAs for BC treatment, it is essential to focus on the molecular mechanisms linking these ncRNAs with iron and ROS metabolism. Further research is also required to understand the relationship between ferroptosis and other regulated cell death pathways, such as TP53-mediated apoptosis, as well as their upstream mechanisms. The role of iron-independent redox processes in ferroptosis must also be explored. Moreover, the lack of specific markers for identifying ferroptosis in living cells and intact tissues hinders the precision of ferroptosis-based treatments and may lead to unwanted side effects. Therefore, identifying specific markers to detect ferroptosis *in vivo* is crucial. For instance, a comprehensive analysis of PLK using databases like Oncomine, GEPIA, cBioPortal, and Kaplan-Meier plots revealed that PLK1 and PLK4 are potential targets for precision treatment in BC, while PLK2, PLK3, and PLK5 may serve as new prognostic biomarkers (Jiawei et al., 2022). Furthermore, studying the ncRNA network in cancer is essential, as dysregulation of this network can inhibit ferroptosis and promote tumor cell survival and progression. Enhancing the detection of circulating lncRNAs and improving diagnostic accuracy remains a key research direction (Schlosser et al., 2016). In addition, some drugs have been shown to regulate ncRNA expression, thereby influencing ferroptosis and exhibiting anti-cancer effects. Hence, identifying and targeting specific ncRNAs while minimizing side effects is an important area for future research. For example, Circ_0069094 enhances the sensitivity of paclitaxel (PTX) in BC by targeting and silencing the miR-136-5p/YWHAZ axis, thus regulating the malignant phenotype and paclitaxel resistance of BC cells (Kong et al., 2023). Traditional herbal medicines, along with their active ingredients and molecular mechanisms, can be identified using public databases. This approach provides a scientific foundation for the prevention and treatment of BC using traditional Chinese medicine. For example, Ecliptae Herba, known for its anti-tumor properties, has been shown through network pharmacology and cytology experiments that TGF-β1 may be a key therapeutic target, with the TGF-β1/Smad signaling pathway playing a critical role (Li et al., 2023). Moreover, nomograms or predictive models for pathologic complete response (pCR) or tumor size reduction could help identify patients likely to benefit from neoadjuvant chemotherapy (NAC) for TNBC, enabling personalized treatment strategies (Yan et al., 2020). Overall, ncRNAs hold significant potential as risk genes, diagnostic markers, prognostic indicators, nanoparticle cargos, and therapeutic targets for BC. Understanding the functions of ncRNAs in future studies may enhance our understanding of breast cancer’s pathogenesis and lead to more efficient, rapid, and precise diagnostic and therapeutic approaches.
